# Development and characterisation of a novel complex triple cell culture model of the human alveolar epithelial barrier

**DOI:** 10.1007/s44164-024-00075-2

**Published:** 2024-08-06

**Authors:** Sarah M. Mitchell, Kirsty Meldrum, Joshua W. P. Bateman, Teresa D. Tetley, Shareen H. Doak, Martin J. D. Clift

**Affiliations:** 1 In Vitro Toxicology Group, Faculty of Medicine, Health and Life Sciences, Swansea University Medical School, Swansea University, Sketty, Wales SA2 8PP UK; 2 Lung Cell Biology, Section of Pharmacology and Toxicology, Airways Disease, National Heart & Lung Institute, Imperial College London, London, UK

**Keywords:** In vitro, Multi-cellular system, Epithelial cells, New approach Methodologies (NAMs), Alveolar, 3D cell culture

## Abstract

Owing to increased pressure from ethical groups and the public to avoid unnecessary animal testing, the need for new, responsive and biologically relevant in vitro models has surged. Models of the human alveolar epithelium are of particular interest since thorough investigations into air pollution and the effects of inhaled nanoparticles and e-cigarettes are needed. The lung is a crucial organ of interest due to potential exposures to endogenous material during occupational and ambient settings. Here, an in vitro model of the alveolar barrier has been created in preparation for use in the quasi-air liquid interface (qALI) and (aerosol) air–liquid interface (ALI) exposures. The model consists of an alveolar type 1-like cell line (TT1), an alveolar type 2-like cell line (NCI-H441) and a model of (alveolar) macrophages (dTHP-1). The model formulates a complex, multi-cellular system, cultured at the air–liquid interface, that mimics the apical layer of the alveolar epithelial region in the human lung. Characterisation data has shown that both TT1 and NCI-H441 epithelial cells are able to be cultured together in addition to dTHP-1 cells through imaging (morphology), pro-inflammatory response and viability measurements. This dataset also demonstrates evidence of a reasonable barrier created by the cell culture in comparison to negative controls. Furthermore, it shows that while maintaining a low baseline of (pro)-inflammatory mediator expression during normal conditions, the model is highly responsive to inflammatory stimuli. This model is proposed to be suitable for use in toxicology testing of inhaled exogenous agents.

**Supplementary Information:**

The online version contains supplementary material available at 10.1007/s44164-024-00075-2.

## Introduction

Inhalation is the primary route of exposure to multiple anthropogenic sources such as inhaled medicines, particulate matter from various sources, e.g. diesel exhaust fumes, and nicotine delivered through tobacco smoke or vaping. Testing the toxicity of inhaled compounds and elucidating the mechanisms surrounding their hazards is particularly complex due to the equally complex nature of the epithelial surfaces on which the compounds make contact. The respiratory tract ultimately terminates at the alveoli, where gas exchange takes place, making the alveolar epithelium a crucial point of biological interest [1]. When damaged through exposure to occupational hazards, pollution or smoking, the alveoli may enter a life-limiting disease state, such as emphysema [2].

The Organisation for Economic Co-operation and Development (OECD) is a group of governmental representatives from 36 countries worldwide. The aim of the OECD is to harmonise policies including those defined for testing and assessment of industrial chemicals, medicines and consumer products. One such policy is that which provides guidance on the development and use of in vitro methods for regulatory use [3]. The guidance document acknowledges various requirements that should be met to generate an effective and reliable in vitro model, including quality assurance, controls and cell culture methodology. The models created and tested in this paper have been generated with the aim of meeting requirements set out within the Good In Vitro Method Practices (GIVIMP) document, which initiated with cell type selection.

The alveolar epithelium consists of two epithelial cell types, namely type I and type II, which both contribute to the function of the alveolar epithelial barrier. Type I cells are squamous and thin and make up roughly 97% of the total surface area of the alveolar epithelium, and although type II cells are the majority of cells in number, type II pneumocytes form only the remaining 3% of the surface area [4]. Despite covering a minimal area compared to type I cells, the type II cells play a key role in protecting normal lung function. Type II cells generate surfactant proteins and, in response to damage, are the progenitor cells of type I cells [5]. Surfactant forms a thin (0.1 µm) layer of liquid on top of the epithelial cells that reduces surface tension and prevents collapse of the lungs during respiration [6]. In addition to the barrier cells, found on top of the alveolar epithelial cells are macrophages which are essential in the response to inhaled toxicants. They are known to be the first cellular defence of deposited exogeneous agents into the lower lung. Macrophages are further vitally important in clearing the alveoli of inhaled material and play a pivotal role in maintaining tissue homeostasis and generating inflammatory reactions [7]. Since the normal function of both epithelial cell types and macrophages within the alveolar epithelium are pivotal for normal respiratory function, it is therefore the focus of this newly characterised model.

Since the introduction of an animal testing ban on cosmetics and their ingredients in 2009 [8], the need for relevant in vitro models, including those mimicking the alveolar region has increased. There are some well-known in vitro models of pulmonary epithelial cells, cell lines such as A549, 16HBE14o^−^, Calu-3 and NCI-H441 cells. Many of these have been characterised as models of individual cells found within the alveolar epithelial barrier, or as part of a multi-cellular model [9]. A number of cellular models have been used in testing responses to inhaled substances [10]. Cellular models such as these have also been exposed to chemicals via submerged (liquid–liquid interface, LLI) and air–liquid interface (ALI) approaches, in order to replicate normal physiology in the lung [9, 11]. Although the addition of multiple cell types, such as epithelial cells and macrophages within one system has aimed to improve in vitro models further [12], a key aspect lacking from these commonly used models is the combination of the two epithelial cell types found at the alveolar epithelial barrier. Even though two barrier cell types form the alveolar epithelium and contribute essential characteristics to the function of the epithelium, there are limited studies published combining and characterising two epithelial cells in culture together to create a model of this. One such study has combined types I and II cell lines (hAELVi and NCI-H441, respectively) and studied the morphology, barrier properties using electrical resistance measurements and genetic screening [13]. The model demonstrated expression of phenotypically relevant genes and barrier properties; however, cells were seeded in a 1:1 ratio, without cell characterisation reported. The lack of characterisation may pose a risk that the model is not in proportion relating to the number, or area, of cells in the human lung, and interpretation of results gained using this model may be difficult to meaningfully understand [12]. Importantly though, Boland et al. showed that the two cell types can be cultured effectively together.

The aim of this study was, therefore, to create and characterise a novel model of the human alveolar epithelium, a constituent of two epithelial cell types, combined with alveolar macrophages. It was also envisioned that the model could be used in submerged and air–liquid interface conditions relevant to exposures, in situ*.* Herein, this study documents the first characterisation of a triple cell culture model containing two alveolar epithelial cell types with macrophage cells. The characterisation of TT1 and NCI-H441 cells in co-culture together with differentiated THP-1 cells in both LLI and ALI conditions is specifically described. Morphology, barrier function and baseline inflammatory response have underpinned the foundation of characterisation of this triple cell co-culture. Through the use of the OECD GIVIMP guidance, the new co-culture can be proposed as a suitable model for testing the toxicity of inhaled substances at both LLI and ALI conditions.

## Methods and materials

### Chemicals and reagents

All chemicals and reagents were purchased from Sigma-Aldrich, UK, unless otherwise stated. The NCI-H441 (HTB-174™) and THP-1 (TIB-202™) cell lines were purchased from the American Tissue Culture Collection (ATCC, USA). After purchase, NCI-H441 and THP-1 cells were used up to passages 30 and 25, respectively. Transduced type-1 (TT1) cells were kindly donated by Professor Teresa Tetley at Imperial College London and used up to a total passage number of 60. TT1 and NCI-H441 cells are used here as models of both type I and type II alveolar epithelial cells, respectively. THP-1 cells are a human, peripheral and monocytic cell line [14] that, once differentiated, are used here as a model of alveolar macrophages.

### Cell culture and seeding

**Fig. 1:**
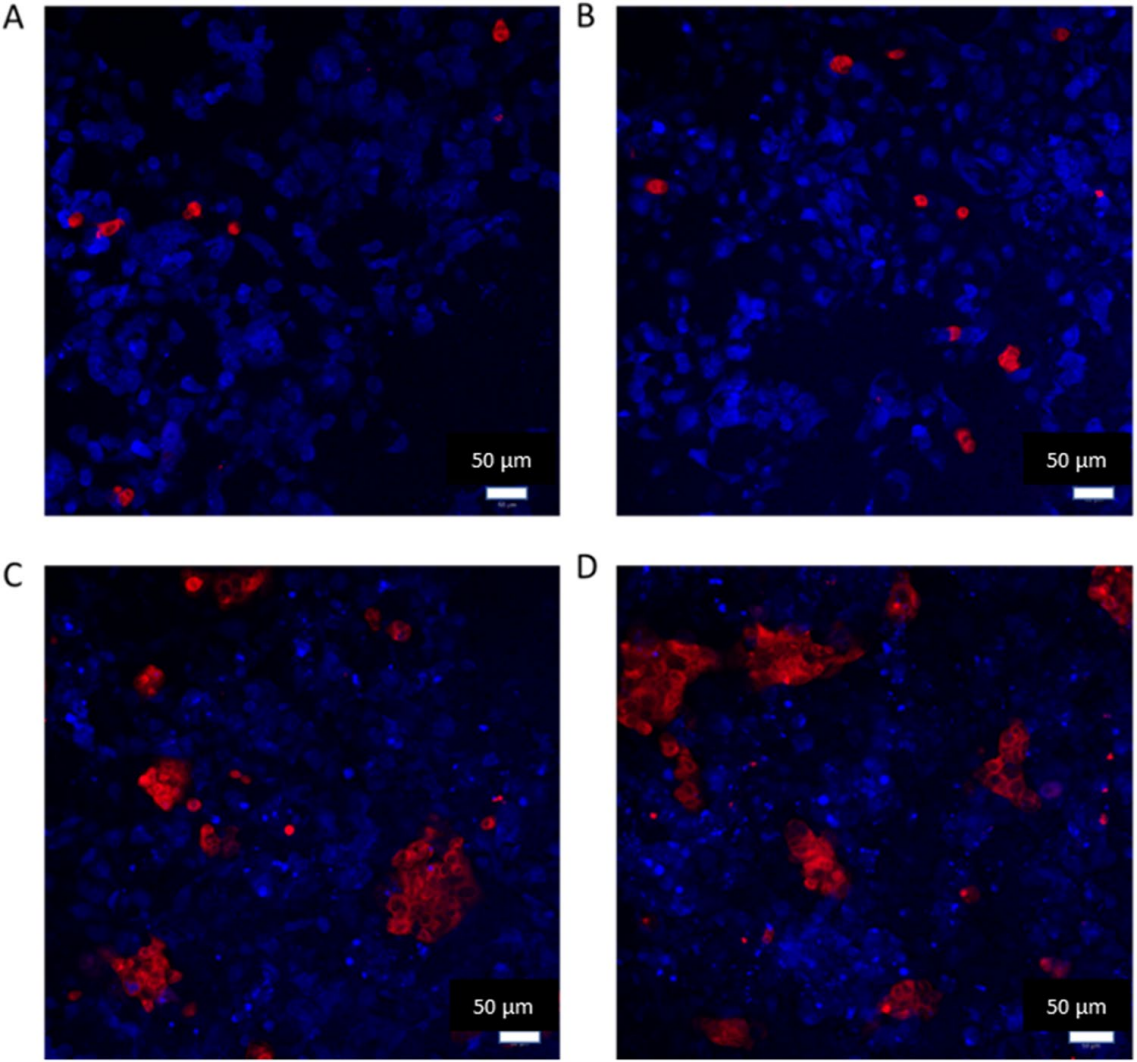
TT1 (blue) and NCI-H441 (red) cells were imaged using CellTracker™ dyes and imaged using confocal microscopy. Cell cultures were imaged in different media ratios and cell seeding densities. Cell cultures imaged are shown with either 1:1 (**A**, **B**) or 11:1 (**C**, **D**) cell seeding ratios, TT1:NCI-H441, respectively. Cultures were submerged in either 50:50 TT1:NCI-H441 media (**A**, **C**) or 100% TT1 cell culture media (DCCM-1) (**B**, **D**). All images were taken after 72 h in culture at × 20 magnification and are representative of *n* = 3

Cells were routinely cultured in T75 flasks, with media changed every 48 h, and cells were passaged when 80–85% confluency was reached. Cells were checked daily using a light microscope for morphology and sterility. Once confluence was reached, cells were seeded into the inserts of 12-well transwell plates (Falcon, UK, 3-µm pore size, 0.9-cm^2^ growth area) after counting using a haemocytometer. The volume of media added apically was 500 µL and basally beneath the insert was 1.5 mL. Various densities were investigated for both cell types to optimise each type. The results of the TT1 and NCI-H441 monoculture seeding optimisation can be found in supplemental data file S1. Different seeding densities were also trialled to gain a biologically relevant density and area coverage of each cell type in co-culture (Fig. 1). All culture media contained 100 U/mL penicillin and 100 µg/mL streptomycin (Gibco, USA). Since TT1 and NCI-H441 cells were routinely cultured in different media types (TT1:DCCM-1 with newborn calf serum (NCS) and NCI-H441:RPMI with foetal bovine serum (FBS)), a brief optimisation to identify the most relevant media type was conducted. The optimisation included measuring co-culture growth and pro-inflammatory response in DCCM-1, RPMI or a 50:50 mixture of both types (supplemental data file S2). Triple cell cultures were created using the finalised co-culture seeding protocol followed by the addition of 100 µL differentiated THP-1 cells (dTHP-1) at a density of 1 × 10^5^ cells per mL (Table 1). THP-1 cells were differentiated using phorbol 12-myristate 13-acetate (PMA). THP-1 cells were incubated with PMA (20 µM) for 48 h. After the incubation period, THP1 cells that had successfully differentiated (dTHP-1) would adhere to the flask. PMA-containing media was removed from the flasks, and fresh culture media was added. Flasks were then incubated for a recovery period of 24 h. After the recovery period had passed, dTHP-1 cells were added to the co-culture which had 70 h of submerged growth. The triple cell culture was then incubated for 2 h to allow dTHP-1 cell adhesion. After incubation, apical media was removed, and the triple cell culture was then incubated for a further 24 h at the ALI.

**Table 1:** Specific cell culture protocol determined for optimal seeding of triple cell co-culture

Cell type	Seeding density (cells/mL)	Co-culture media	Time cells added to wells	ALI time point	Exposure time point
TT1	4.58 × 10^5^	100% DCCM-1	0 h	72 h	96 h (24 h ALI)
NCI-H441	4.16 × 10^4^		0 h		
dTHP-1	1 × 0 10^5^		70 h		

### Cell counting and viability

Cell culture viability was measured using trypan blue exclusion, frequently, throughout the protocol development process. To measure viability, cells were first removed from inserts using trypsin. Cells were then pelleted in 15-mL tubes using a centrifuge and re-suspended in culture media. A 1:1 solution of cell suspension and trypan blue solution was mixed thoroughly; then, the solution was added to a haemocytometer where live (unstained) and dead (stained, blue) cells were counted, allowing calculation of percentage viability.

### Exposure to carbon black

Cell cultures were exposed to carbon black (CB) either at ALI or qALI. To prepare CB suspension, 8.9 mg CB was pre-wetted with 25 µL ethanol (100%) before being made up to 2.6 mL using distilled water forming a 3.4-mg/mL suspension. The suspension was sonicated using a Branson SFX 550 Sonifier Kit at 10% power, at intervals of 10 s on and 10 s off for a total sonication time of 4 min. Stock CB suspension was then diluted to the final concentration using distilled water.

ALI exposures were conducted using a VitroCell Cloud12, following the method previously described by Bannuscher et al. (2022) [15]. Briefly, CB suspensions were spiked with 0.009% NaCl to assist nebulisation with deposited concentrations of 390, 780 and 3100 ng/cm^2^ targeted. Comparable CB concentrations of 1.64, 3.28 and 13.04 µg/mL were prepared in sterile distilled water and exposed at qALI by adding 21.7 µL of CB suspension directly to the apical membrane. Cultures were then exposed for 24 h before endpoint assessment.

### Exposure to DQ_12_ particles

DQ_12_ is a reference sample of quartz (crystalline silica) at < 5 µM in size [16]. DQ_12_ is known to induce inflammatory responses from both macrophage and epithelial cells [17]. For these reasons, DQ_12_ is used here as a positive control for inflammation, as well as a particle control for comparison to carbon black. High (10 µg/cm^2^) and low (1 µg/cm^2^) concentrations of DQ_12_ particles were suspended in complete cell culture media and thoroughly dispersed by sonication. Similarly to carbon black, DQ_12_ (21.7 µL) was added directly to the apical membrane for 24 h.

### Epithelial barrier integrity

Trans-epithelial electrical resistance (TEER) was used to measure barrier tightness throughout this study for submerged cultures only. The TEER machine (World Precision Instruments, UK) used a chopstick electrode which was placed with one electrode inside the apical region, and the second electrode in the basal region of the insert. A current was then passed through the culture medium and total resistance was recorded. After cells were seeded, TEER measurements were taken every 24 h, from four different areas within each well (as previously described in Bannuscher et al. [15]). The four results from each well were averaged, and the baseline resistance generated from the insert was subtracted. Importantly, TEER measurements were only conducted with submerged cultures.

To measure barrier integrity in both cell cultures in submerged conditions, and at the air–liquid interface, the translocation of blue dextran was used. Once ready for testing, basal media was replaced with new media, and apical media was removed, where applicable. Then, 250 µL of a 2.5 mg/mL solution of blue dextran was added to the apical side of the insert. The inserts were then incubated for 2 h before removing basal media and placing 100 µL into 96-well plates. The basal media was then analysed at 600 nm using a plate reader (BMG Labtech Ltd, UK). As a positive chemical control, Trypsin–EDTA (10 nM) was used in order to create a permeabilised epithelial barrier.

### Qualitative and morphological imaging

Cell cultures were often viewed under a light microscope, as well as a fluorescent microscope to visualise cell morphology. After cells were seeded in transwell plates, cells were fixed in preparation for fluorescent staining every 24 h using 4% paraformaldehyde (PFA). Cell cultures were then stained using either morphological stains (DAPI, phalloidin, CD11b) or qualitative stains (CellTracker™ (Violet BMQC, C10094 and Deep Red, C34565), Invitrogen, UK). The phalloidin solution with Alexafluor 488 (Molecular Probes, Invitrogen AG, Basel, Switzerland) was made up to a 1:50 dilution, and 6 µL of Vectashield (Vector Laboratories, USA) containing DAPI stain was used per slide. CellTracker™ dyes were used to confirm the presence of both epithelial cell types. CellTracker™ Violet BMQC (C10094, Invitrogen, UK) and CellTracker™ Deep Red (C34565, Invitrogen, UK) were used for TT1 and NCI-H441 cells, respectively. The supplier’s methods were followed, but briefly, TT1 and NCI-H441 cells were incubated separately with CellTracker™ Violet BMQC (20 µM, 30 min) and Deep Red (10 µM, 30 min), respectively. Cell cultures were then viewed using confocal microscopy (ZEISS, Germany, LSM710).

### (Pro)-inflammatory concentration measurement

Cell culture supernatants were collected from all cell culture types, every 24 h after seeding for analysis of (pro)-inflammatory mediators (interleukin (IL)-6, (IL)-8). Levels of secreted IL-8 and IL-6 were determined using enzyme-linked immunosorbent assay (ELISA) kits (Human IL-8/CXCL8 DuoSet (catalogue no. DY208) and Human IL-6 DuoSet (catalogue no. DY206), R & D Systems, USA) as per the manufacturer’s guidelines.

### Data and statistical analysis

Data and statistical analysis were determined and created in GraphPad Prism, version 9.3.1. Statistics used were always a one-way ANOVA unless otherwise stated, followed by Dunnett’s post hoc test where significance (*p* < 0.05) was seen in the ANOVA. Confocal fluorescent images were captured using Zen Black (v.2.3 SP1, Carl Zeiss, UK) software, and light microscope images were captured using Zen Blue (v.2.3 Lite, Carl Zeiss, UK) software. All data is *n* = 3 (in triplicate) unless otherwise stated.

## Results and discussion

### Growth and viability of cell cultures

TT1 and NCI-H441 cells were initially characterised in monocultures at the ALI before adding both cell types together to understand the baseline behaviours of each cell type in both mono- and co-culture formats (supplemental data file S1). Subsequently, to create a biologically relevant co-culture of type I- and type II-like cells, particular attention was paid to the ratio of each cell type to one another in the human lung. Firstly, cells were seeded in different ratios as dictated by the quantities of alveolar type I and II cells within the total tissue fraction, which were previously recorded as 8.3 ± 0.06% and 15.9 ± 0.8%, type I and II, respectively [18]. Based on this literature, a seeding density of 1:2 would be applicable; however, TT1 cells were observed to cover a smaller area than NCI-H441 cells, which would lead to a culture where type II cells occupy less area than that of type II cells in situ. Therefore, in an attempt to replicate the ratio of cells in vivo*,* based on growth rates and growth area, a seeding ratio of 1:1 TT1 to NCI-H441 cells in complete DCCM-1 media or 50:50 DCCM-1 and RPMI was trialled. To elucidate which culture environment was ideal to construct the co-culture further, cell cultures were imaged using live CellTracker™ staining, as shown in Fig. 1A and B.

**Fig. 2:**
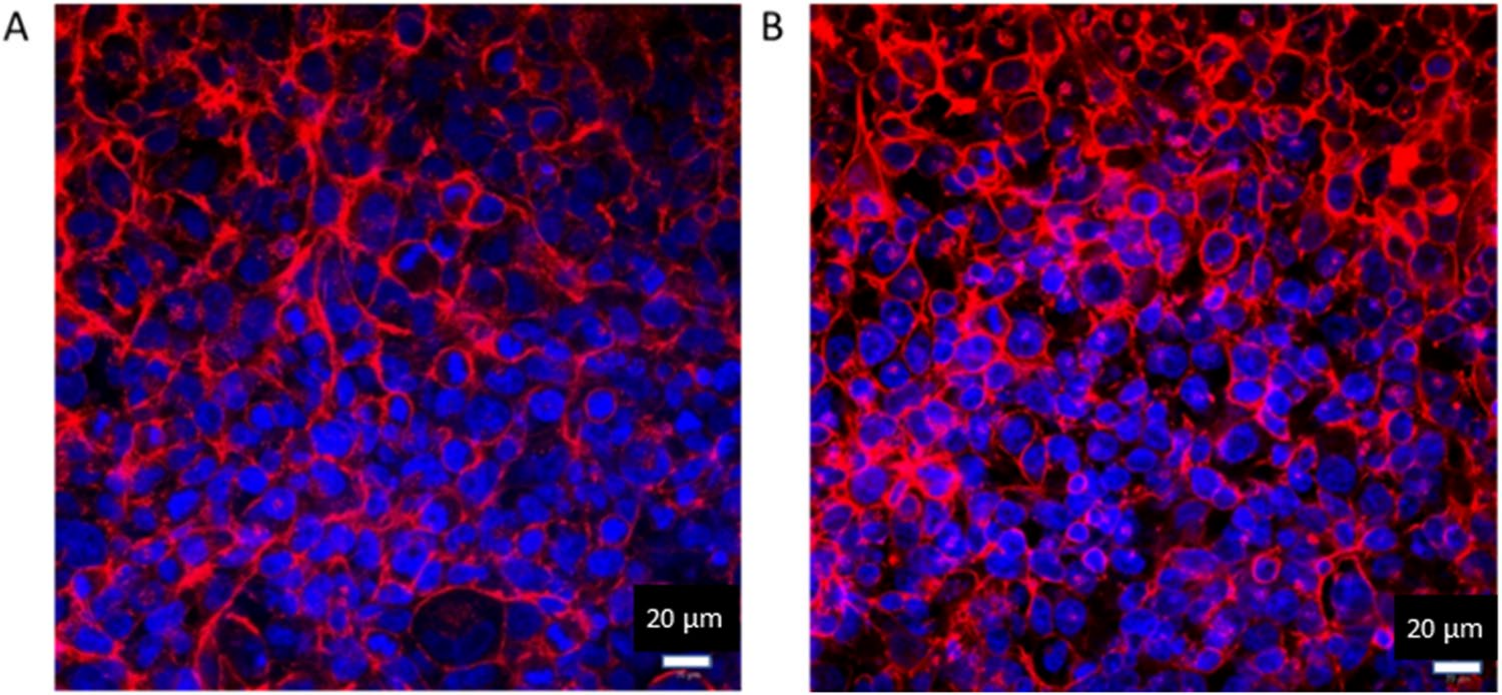
Images recorded using confocal microscopy of co-cultured TT1 and NCI-H441 cells seeded at an 11:1 ratio, respectively. Images were taken after 24 h (**A**) and 48 h (**B**) of culture at the ALI. Stains used were DAPI (nuclei, blue) and phalloidin (actin filaments, red). All images were taken at × 40 magnification, and all images are representative of *n* = 5

**Fig. 3:**
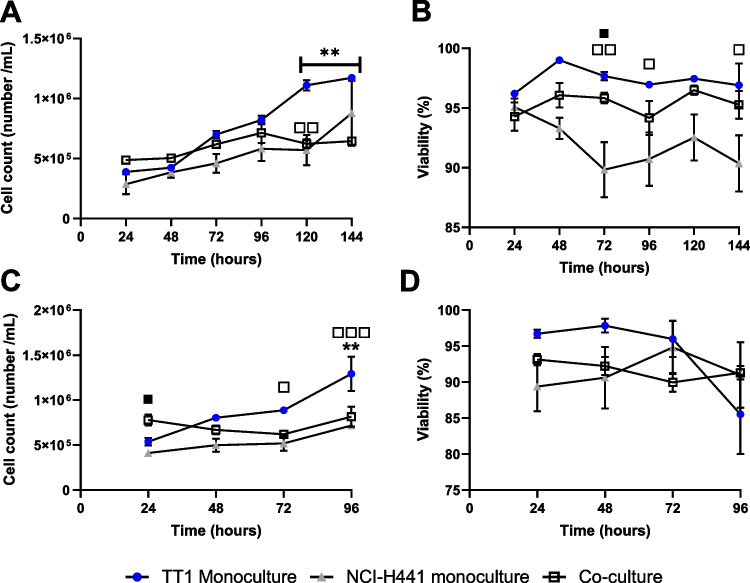
Cell number and viability of TT1 and NCI-H441 co-cultures compared to TT1 and NCI-H441 monocultures. Datasets (**A**, **B**) were recorded in submerged conditions and (**C**, **D**) in ALI conditions. Data was calculated using trypan blue exclusion assay every 24 h after seeding. Co-culture was seeded at an 11:1 ratio, TT1:NCI-H441, respectively, TT1 cells seeded at 5 × 10^5^ cells/mL and NCI-H441 seeded at 2.5 × 10.^5^. Data shown are *n* = 3, ± SEM. Statistical significance is noted using asterisks (*) when comparing TT1 monoculture with co-culture in 100% DCCM, white square (**□)** when comparing TT1 monocultures with NCI-H441 monocultures and black square (**■**) when comparing NCI-H441 monocultures and co-cultures. The number of significance symbols defines the level of significance; * *p* < 0.05, **p < 0.01

Differences in the media type and components of complete media had minimal effects on how the cells were cultured together; however, the 1:1 seeding density was found to be unsuitable based on the low coverage of NCI-H441 cells (Fig. 1A, B). Further, NCI-H441 cells were observed to have a slower growth rate than TT1 cells (Fig. 3). Therefore, a trial of seeding NCI-H441 cells prior to adding TT1 cells occurred. It was found that seeding NCI-H441 cells early was not effective. During this trial, the NCI-H441 cells occupied most of the surface area, rendering the TT1 cells unable to grow alongside the NCI-H441 cells in a monolayer. Cells were then seeded based on surface area coverage in the human alveoli, which has previously been reported as 92.9 ± 1% for type I and 7.1 ± 1% for type II [18]. Based on this data, cells were seeded at an 11:1 ratio of TT1 to NCI-H441, respectively, in an attempt to create a similar level of surface area coverage of each cell type as in vivo. Images of the cells cultured together confirmed that both cell types were present and successfully viable (Fig. 3B, D) in culture after 72 h, as well as showing relevant area coverage (Fig. 1C, D). Usually in other studies combining multiple cell types, specific cellular labels are used for imaging to show the presence of different cell types [19], or have measured and compared overall gene expression in monocultures and co-cultures [20]. In this work, both cell types were known to express many of the same phenotypic markers due to their function as alveolar epithelial cells. Thus, this alternative staining approach was used. A downfall of using CellTracker™ is that as cells proliferate the dye within the cell cytoplasm is depleted, and therefore, areas without staining are evident. Therefore, to confirm a confluent monolayer, images were also captured using actin filament and nuclei stains via confocal microscopy (Fig. 2). At 24 h (Fig. 2A), a large proportion of the growth area is already occupied, and at 48 h (Fig. 2B), cells appear to be compressing and forming a confluent monolayer with minimal growth area remaining.

Based on the findings of this methodological approach to formulate the co-culture of type 1 and type 2 alveolar epithelial cells, the final seeding density for monocultures of TT1 cells was 5 × 10^5^ cells/mL, and NCI-H441 cells were 2.5 × 10^5^ cells/mL. The optimal seeding density used for co-cultures was a total number of cells of 5 × 10^5^ cells/mL, with a ratio of 11:1, TT1 to NCI-H441 cells, respectively (4.58 × 10^5^ cells/mL for TT1 cells and 4.16 × 10^4^ cells/mL for NCI-H441 cells). The different seeding densities of each monoculture and co-culture are detailed in Table 2.

**Table 2:** Final cell seeding densities for each culture type after characterisation both in cells/mL and per cm^2^ growth area

Culture type	TT1	NCI-H441
TT1 monoculture	5 × 10^5^ cells/mL (2.25 × 10^5^/cm^2^)	None
NCI-H441 monoculture	None	2.5 × 10^5^ cells/mL (1.125 × 10^5^/cm^2^)
TT1 + NCI-H441 co-culture	4.58 × 10^5^ cells/mL (2.061 × 10^5^/cm^2^)	4.16 × 10^4^ cells/mL (1.86 × 10^5^/cm^2^)

**Fig. 4:**
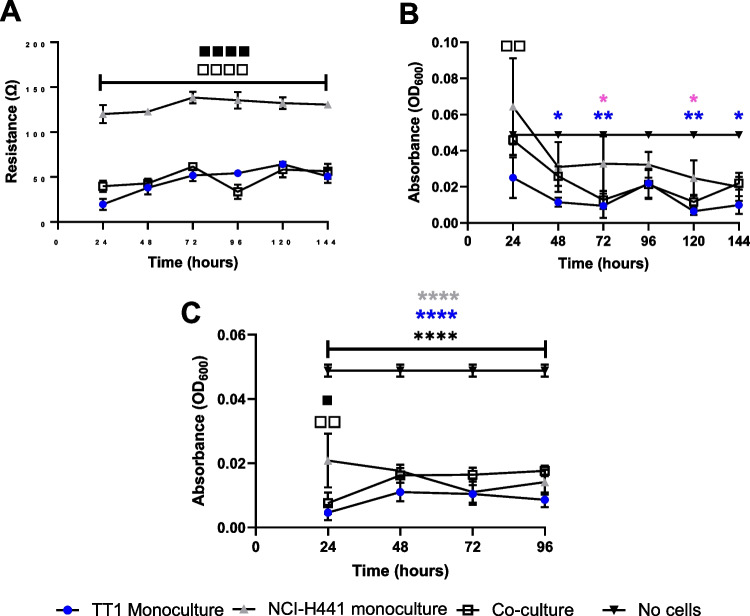
Barrier properties of cultured cells were measured after co-cultures were seeded at 11:1 seeding density (TT1:NCI-H441, respectively), TT1 monocultures seeded at 5 × 10^5^ cells/mL and NC-H441 monocultures seeded at 2.5 × 10.^5^ / mL. TT1 + NCI-H441 co-cultures were statistically compared to TT1 and NCI-H441 monocultures, in both submerged conditions (**A**, **B**) and ALI conditions (**C**). Measurements were taken every 24 h using blue dextran absorbance (**A**, **C**) and trans-epithelial electrical resistance (TEER) (**B**). Data shown are mean values of 5 biological replicates (*n* = 5), ± SEM. For graph (**A**), significance is noted using white square (□) when comparing TT1 monocultures with NCI-H441 monocultures and black square (■) when comparing NCI-H441 monocultures and co-cultures. For graph (**B**), significance is noted using blue asterisk (*) and pink asterisk (*) symbols when comparing TT1 monocultures and NCI-H441 monocultures with no cells, respectively. For graph (**C**), blue asterisks (*), grey asterisks (*) and black asterisks (*) are used when comparing TT1 monocultures, NCI-H441 monocultures and co-cultures with no cells, respectively. The number of significance symbols defines the level of significance; *p < 0.05, **p < 0.01, ****p < 0.0001

Focussing on the 11:1 ratio, an assessment of cell growth, viability and inflammatory status was conducted. All of these were completed in different cell culture media types (as each cell has different media), since it is important to make sure that each cell type remains correct in terms of structure and function within a co-culture, as well as to get the cell:cell ratio correct. For example, previous works have identified that different levels of serum and components within the media contributed significantly to overall culture viability and growth [21]. Here, serum concentration levels were the same for each cell type–specific media, but the type of serum was different (i.e. FBS and NCS), so it was important to compare the two and understand any potential negative biological impact they could have upon the structure and function of each epithelial cell type, due to this difference. After reviewing the (pro)-inflammatory status of co-cultures seeded in both media types (supplemental data S3), there was no increase in the release of IL-6 or IL-8, and therefore, due to the number of TT1 cells to NCI-H441 cells, 100% DCCM-1 was chosen for optimal conditions. Based upon these findings using the confirmed final seeding density (11:1) and culture media (100% DCCM-1), baseline cell number, viability (Fig. 3) and barrier integrity (Fig. 4) were recorded in both submerged and ALI conditions. The count and viability of co-cultured cells in optimised conditions are shown in supplemental data file S4.

Figure 3 shows TT1 monocultures continue to grow actively at later time points, and significantly (*p* < 0.05) more than both the NCI-H441 monocultures and TT1_NCI-H441 co-cultures, up to 120 h under submerged conditions. Similar findings were also observed in ALI conditions when comparing TT1 cells with NCI-H441 monocultures and the co-culture at 96 h. These findings were expected due to the higher doubling rate of TT1 cells (20 h) compared to the NCI-H441 cells (58 h). NCI-H441 cells, when cultured under submerged conditions, were noted to have a significantly (*p* < 0.05) lower viability than both TT1 monocultures and TT1_NCI-H441 co-cultures after 72 h in culture (Fig. 3B). Any further significant (*p* < 0.05) differences noted in viability (Fig. 3B) were always seen when comparing NCI-H441 and TT1 cell cultures. Though there are significant differences between culture types, the overall viability of each culture type continuously remained above 88% and therefore was not identified as an issue. Interestingly, when culturing both cell types at the ALI, either as mono- or co-cultures, viability remained similar (Fig. 3D). Cell number data in submerged conditions (Fig. 3A) show the most similarities between the co-culture and NCI-H441 cells, but the most similarities in viability (Fig. 3B) are seen between TT1 and co-cultures. Then at the ALI, significant differences (*p* < 0.05) fluctuate between cell culture types (Fig. 3C, D). This phenomenon confirms that there are two different cell types present and that they are both contributing different characteristics when in co-culture at the ALI.

### Barrier integrity of cell cultures

Barrier tightness was measured using both TEER and blue dextran for submerged cultures, and blue dextran only for ALI cultures (Fig. 4). Data was recorded initially on monocultures and then repeated in co-culture. The data from each cell culture type were then compared. No significant differences (*p* > 0.05) were noted between submerged TT1 monocultures and co-cultures for TEER. NCI-H441 cells, on the other hand, formed a significantly (*p* < 0.05) more resistant barrier than both TT1 cells and co-cultures. Between 48–72 h and 120–144 h, TT1 monocultures created a significantly (*p* < 0.05) tighter barrier compared to the negative control (no cells). NCI-H441 monocultures created a significantly (*p* < 0.05) tight barrier compared to the negative control at 72 and 120 h. Surprisingly, in submerged conditions, the co-culture barrier function was not significantly different (*p* > 0.05) from the negative control at any time point, though the dataset appears similar to that recorded in TT1 monocultures. Once at the ALI (Fig. 4C), and after the initial 24 h in ALI culture, differences between TT1 and NCI-H441 monoculture types, as well as between the NCI-H441 monoculture and co-culture, were observed; however, all cell culture types formed a barrier that was significantly (*p* < 0.05) less permeable than the negative (cell-based) control.

Many monocultures and co-cultures created for use as an alveolar model focus largely on the barrier properties and molecular transport through the cell culture due to the role of the epithelium in situ [22]. While significantly (*p* < 0.05) higher resistance values compared to the negative control were measured in all cell types used here, this model does not create a tight barrier when compared to other published data. For example, others have recorded TEER values at far higher levels in cultures of NCI-H441 monocultures (1010 ± 105 Ω/cm^2^ in submerged conditions and a maximum of 315 Ω/cm^2^ in ALI conditions) [23]. However, in these earlier datasets, studies used dexamethasone to encourage strong barrier formation which may explain the lack of tight barrier formation in this study. Dexamethasone was not used in this study due to the model being created for toxicology testing. Previous studies have shown that dexamethasone can be cytotoxic and interfere with inflammatory pathways through a reduction of responses to LPS [24, 25]. Both cytotoxicity and inflammation are crucial endpoints that will be studied after exposure to inhaled toxicants, therefore adding further variables with the risk of synergistic effects is not ideal when considering in vitro mechanistic toxicology studies. However, there are studies that show inter-laboratory differences even when using the same protocols and cell types in both monocultures and co-cultures [15, 26, 27], and therefore, variations in these measurements would be expected. The use of valid controls and repeated measurements can help reduce any negative impacts and maintain the reliability of the model.

### Baseline (pro)-inflammatory response of cell cultures

**Fig. 5:**
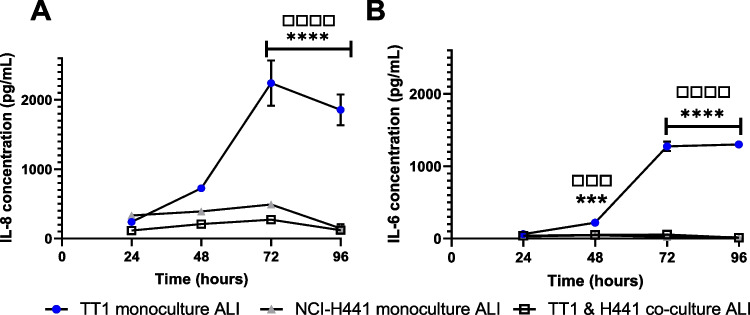
ALI—levels of IL-8 (**A**) and IL-6 (**B**) concentrations in basal supernatants were compared between TT1 monocultures, NCI-H441 monocultures and TT1 + NCI-H441 co-cultures after growth at ALI. Supernatants were taken every 24 h for a total of 96 h after starting ALI conditions. Data shown are *n* = 3, ± SEM. Significance is noted using asterisk (*) symbols when comparing TT1 monoculture with co-cultures and white box (□) symbols when comparing TT1 monocultures with NCI-H441 monocultures. The number of significance symbols defines the level of significance; ***p < 0.001, ****p < 0.0001

Frequent measurements of IL-8 and IL-6 were conducted in both submerged and ALI conditions (Fig. 5) for all cell and culture types. The results from the submerged culture scenarios are described in supplemental data file S3 and Table 3. Quantifying levels of such markers helps to understand the capabilities and baseline culture conditions, especially when changing a cell culture from submerged to ALI conditions or introducing a second cell type to the culture environment. For the ALI culture scenarios, in terms of the IL-8 response (Fig. 5A), it was observed that this chemokine was readily produced by the TT1 monocultures after 48 h (compared to 24 h), significantly (*p* < 0.05) increasing, from baseline, at both 72 h and 96 h. A similar trend was observed in both the NCI-H441 monocultures and TT1_NCI-H441 cells. However, the effect level was limited, with no statistical (*p* > 0.05) or biological significance. A similar trend of effect across the same cell culture scenarios was noted for the IL-6 cytokine. A statistically significant (*p* < 0.05) was observed at 48 h, raising further (> tenfold increase in effect level) at both 72 h and 96 h. Both the NCI-H441 monoculture and TT1_NCI-H441 co-culture indicated limited IL-6 production over the 96-h period (*p* > 0.05). Importantly, the finding that both pro-inflammatory mediators were limited at baseline (i.e. 24-h sample period) suggests that the co-culture model is potentially more reliable than the TT1 monoculture for measuring (pro)-inflammatory responses in vitro in response to exposures.

**Table 3:** Summary of results for co-cultured cells

Characteristic	Summary	Optimal results
Co-culture seeding protocol	Monocultures characterised previously have been combined and assessed for cell proliferation and viability. The ratio of type I to type II cells has been confirmed through the use of physiological data for humans, combined with fluorescent imaging to show the ratio of cells present in culture. Optimal cell culture media was also specified	Seeding ratio: 11:1 for TT1 to NCI-H441, respectively Media: 100% DCCM-1
Confirmed monolayer formation	Fluorescent imaging was used to identify from which point the co-culture was fully confluent	Confluent by 72 h
Barrier function	In submerged conditions, the co-culture did not form a tight barrier. When grown at the ALI, at all measured time points, a statistically significant (*p* < 0.05) tight barrier was formed	Tight barrier formed once co-culture is grown at the ALI
(Pro)-inflammatory status	IL-6 and IL-8 were measured, and co-cultures gave rise to significantly (*p* < 0.05) lower concentrations of both IL-6 and IL-8 in ALI conditions	IL-6 and IL-8 were consistently and significantly (*p* < 0.05) lower in co-cultures

### Triple cell culture

**Fig. 6:**
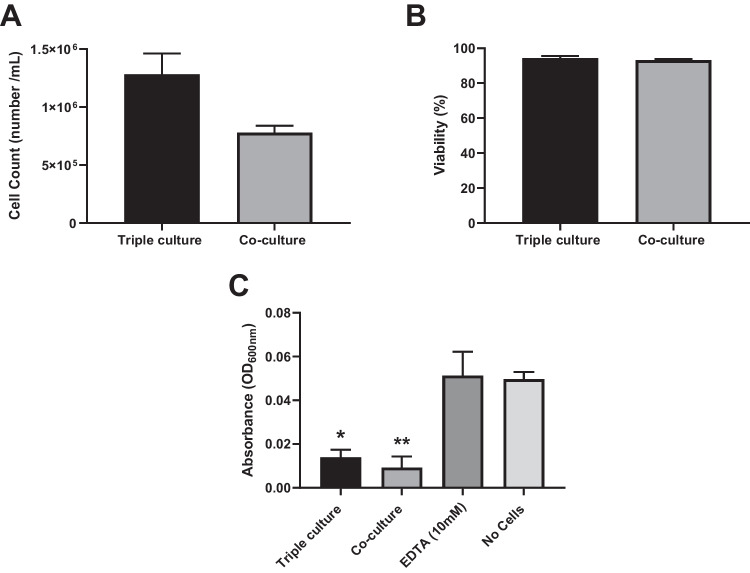
The total cell number (**A**), overall viability (**B**) and barrier integrity (**C**) of co-cultures of TT1 and NCI-H441 cells and triple cell cultures after 24 h at ALI. Cell counts and viability were recorded using trypan blue exclusion assay, and barrier integrity was measured by blue dextran translocation. Data shown is *n* = 3 ± SEM for cell counts and viability and *n* = 5 ± SEM for barrier integrity. Significance is noted by *p ≤ 0.05, **p ≤ 0.01. Statistical significance seen when compared to control data (no cells)

To progress the co-culture further, differentiated THP-1 cells (dTHP-1) were added to the co-culture once at the ALI to create a triple cell co-culture. Similarly to the protocol described in 2022 by Meldrum et al. [28], the dTHP-1 cells were added after 70 h of submerged culture time, and apical media was removed after 2 h which allowed the adhesion of the dTHP-1 cells to the underlying co-culture of epithelial cells. The cell number, viability and barrier integrity were measured from 24 h post-ALI and compared to co-culture results at the same corresponding time point. For a brief characterisation of the new triple cell culture, Fig. 6 shows the expected effect of adding cells, an increased total cell number in culture. Overall, similar levels of cellular viability were observed in both the TT1_NCI-H441 co-culture and the triple cell co-culture. Both triple and co-cultures created a significantly (*p* < 0.05) less permeable barrier when compared to the experimental control of no cells.

**Fig. 7:**
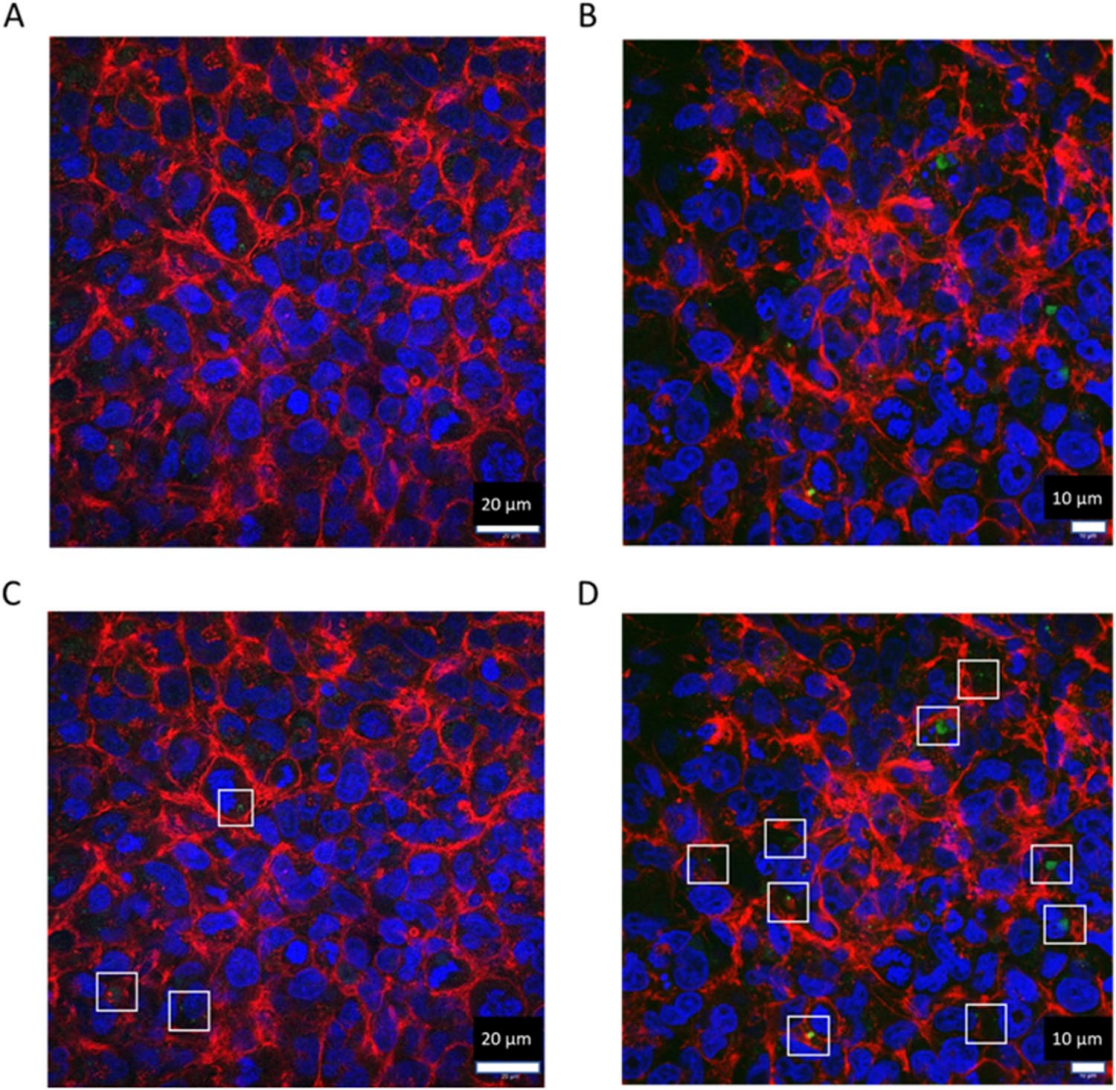
Confocal microscopy images of the triple cell culture, containing TT1, NCI-H441 and dTHP-1 cells. Images were taken at 24 h post ALI at × 40 magnification. Cell cultures were stained using DAPI (nuclei, blue), phalloidin (actin filaments, red) and CD11b (green, macrophage marker). Images are two different areas in culture (**A**, **B**) representative of *n* = 3. Images **C** and **D** are the same image as **A** and **B**, respectively, with CD11b positive staining highlighted in white boxes

**Fig. 8:**
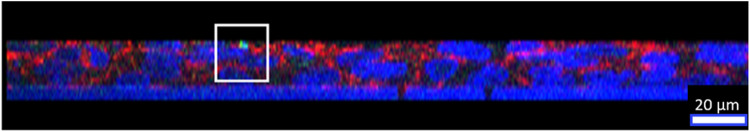
Confocal microscopy image collected from a Z stack in X:Y projection format of the triple cell culture, containing TT1, NCI-H441 and dTHP-1 cells. Cell cultures were stained using DAPI (nuclei, blue), phalloidin (actin filaments, red) and CD11b (green, macrophage marker). Image was taken 24 h post ALI, at × 63 magnification. CD11b stained dTHP-1 cells within triple cell culture are highlighted in the white box. Image is representative of *n* = 3

Although the data showed that adding the third cell type increased cell numbers overall (Fig. 6A), it was not yet shown that macrophages remained within triple cell culture after incubation. Figure 7 shows the results of fluorescent staining in triple cell cultures. Since dTHP-1 cells exhibit different cellular markers compared to the epithelial cells, a monocytic marker was used to confirm the presence of dTHP-1 cells (CD11b) in combination with phalloidin (f-actin) and DAPI (nuclei) to highlight the cell culture overall. Figure 8 shows the same results from Fig. 7, but from an alternative plane as a cross section to see where the macrophages are located.

### Testing the capabilities of the triple cell model

**Fig. 9:**
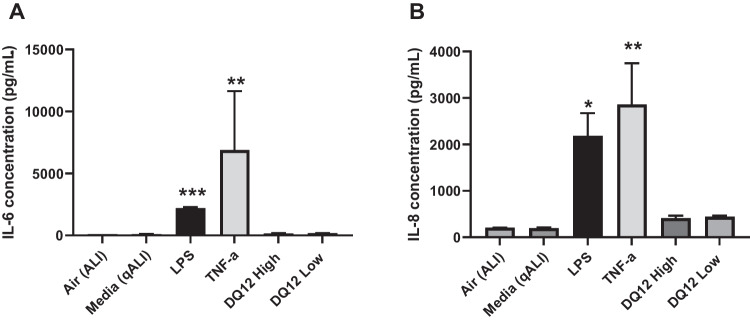
IL-6 (**A**) and IL-8 (**B**) concentrations after exposures to positive controls at the qALI. All data shown is *n* = 3, ± SEM. Significance is noted by *p ≤ 0.05, **p ≤ 0.01, ***p ≤ 0.005. Statistical significance seen when compared to control data (media (qALI))

After characterisation, the new triple cell culture was then exposed to a number of different, known positive chemical and particle controls to assess a pro-inflammatory response in vitro (Fig. 9). The positive controls were all administered at the quasi-ALI (qALI), a method previously described based on area coverage where an ALI exposure was not possible [29]. After exposure, both LPS (Sigma, #L2630-10MG, from *Escherichia coli* O111:B4, 1 mg/mL) and TNFα (Bio-techne, #NBP2-35076-50ug, 10 µg/mL) showed evidence of significantly (*p* < 0.05) elevated IL-6 and IL-8. Neither concentration of DQ_12_ elicited a response; however, this was later confirmed as a consequence of inactive surface chemistry [28]. These data show that the model elicits a measurable and significant (p < 0.05) response to known positive controls for inflammation and therefore can be used in future testing of materials with unknown mechanisms of toxicity.

**Fig. 10:**
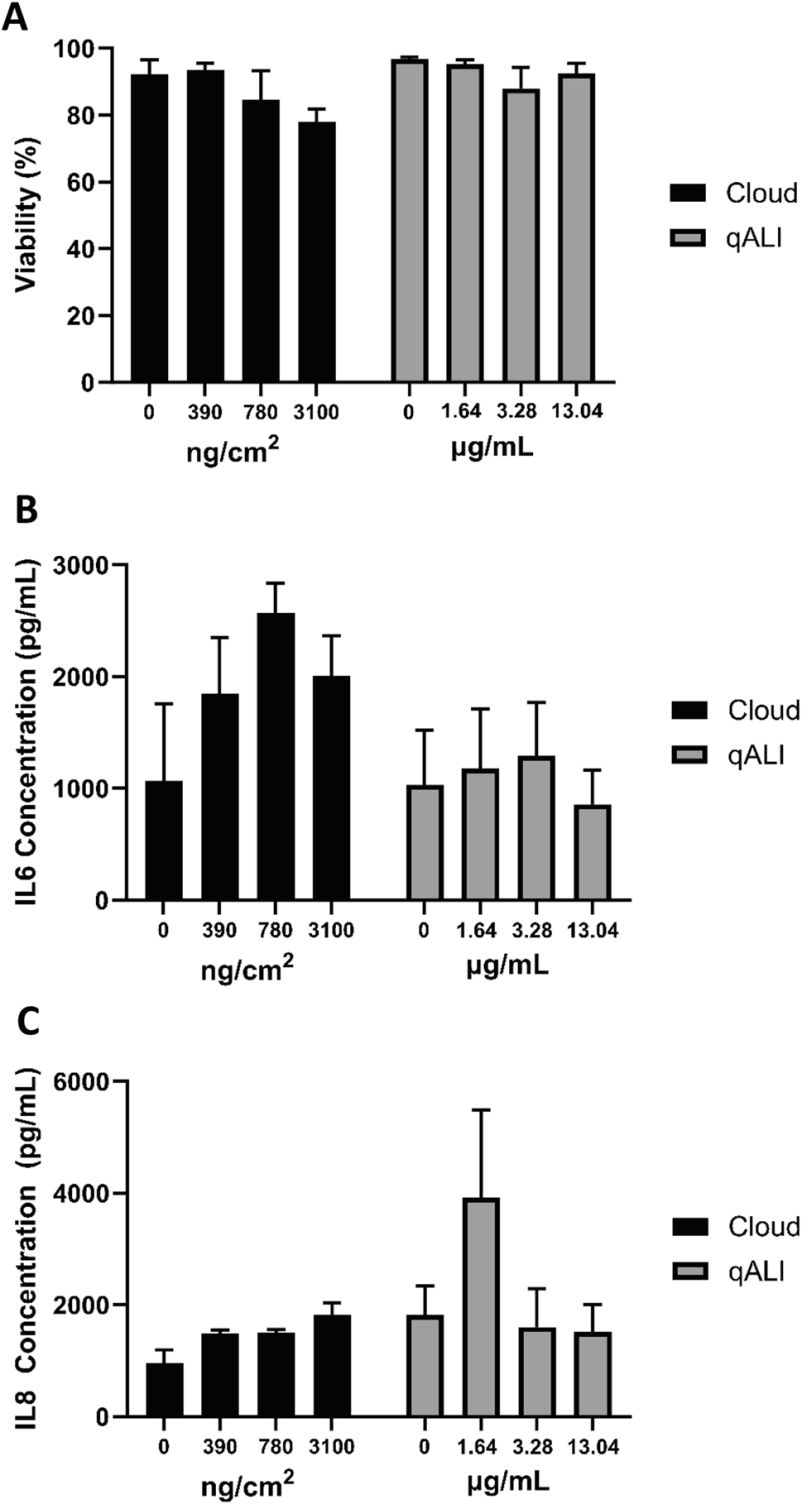
Cytotoxicity (**A**) and IL-6 (**B**) and IL-8 (**C**) concentrations after exposures to CB using the VitroCell Cloud12 or at qALI. All data shown is *n* = 3, ± SEM

The triple cell co-culture was also exposed to Printex90 carbon black (CB) at the ALI using a VitroCell® Cloud12 and contrasted with a comparable concentration of CB administered at the qALI. Both ALI and qALI CB exposures resulted in a non-significant (*p* > 0.05) dose-dependent decrease in viability, though this trend was more obvious in the ALI exposures (Fig. 10A). ALI exposures induced a non-significant (*p* > 0.05) increase in the release of both IL-6 and IL-8, though this trend was not as clear in those exposed at qALI, which exhibited a more varied response (Fig. 10B, C). Importantly, the findings observed with the different positive particle and chemical controls further emphasises the need to choose proper positive controls for in vitro models, based upon the physico-chemical characteristics of the samples (i.e. particle controls) and the cell types within the system (i.e. chemical controls).

## Conclusions

Building upon previous data collected from monocultures of each cell type, the triple cell co-culture formed here has been characterised and has shown potential for use in toxicity testing of inhaled compounds. The model includes three cell types: two epithelial cells (TT1 and NCI-H441) as models of type I and II alveolar epithelial cells, respectively, with an inflammatory cell type (dTHP-1) as a model of alveolar macrophages. Characterisation has included measuring key baseline characteristics, including viability, barrier integrity and (pro)-inflammatory response in both submerged and ALI conditions. The model has been tested using known positive controls (Table 4) for (pro)-inflammatory response and air pollution and has proven the ability to respond significantly to inflammatory stimulation. Overall, the triple cell culture provides a responsive and biologically relevant model of the human alveolar epithelium which can be used as an in vitro toxicology testing tool for inhaled xenobiotics.

**Table 4:** Summary of effects seen after exposure to positive controls

Control	Result
LPS (1 mg/mL)	Significantly increased release of both IL-6 and IL-8 after exposure to LPS
TNFα (10 µg/mL)	Significantly increased release of both IL-6 and IL-8 after exposure to TNFα
DQ_12_ high (10 µg/cm₂)	No reaction—due to inactive surface chemistry
DQ_12_ Low (1 µg / cm^2^)	No reaction—due to inactive surface chemistry
Carbon black (1.64–13.04 µg/mL)	Non-significant but dose-dependent changes in viability

## Supplementary Information

Below is the link to the electronic supplementary material.

Supplementary file1 (DOCX 170 KB)

## Data Availability

All data is available upon request to the contact author.
