# Supplementary material for: Development and characterisation of a novel complex triple cell culture model of the human alveolar epithelial barrier
Source: In Vitro Model. 2024 Aug 6;3(2-3):125–37. doi: 10.1007/s44164-024-00075-2 (PMC11756452; doi:10.1007/s44164-024-00075-2)
Supplement: Supplementary file 1 — Supplementary file1 (DOCX 170 KB) [file 44164_2024_75_MOESM1_ESM.docx]

**Supplemental Information**

**Characterisation of TT1 and NCI-H441 monocultures**

Figure S1 - TT1 monoculture characteristics at the ALI. These data represent the baseline characteristics on TT1 cells when seeded at the optimised 5 x 10^5^ cells / mL. Cell doubling **(A)** continued through 96 hrs of culture, viability **(B)** remained above 85% throughout, and barrier integrity **(C)** remained significantly(p<0.05) increased up to 96 hrs when compared to negative controls.

Figure S2 - NCI-H441 monoculture characterisation at the ALI. These data represent the baseline characteristics on NCI-H441 cells when seeded at the optimised 2.5 x 10^5^ cells / mL. Cell doubling **(A)** continued through 96 hrs of culture, peaking at 96 hrs, viability **(B)** remained above 90% and barrier integrity **(C)** remained significantly (p<0.05) increased than negative controls up to 96 hrs.

**Establishing suitable culture conditions for the co-culture**

Table S1 - Different cell number ratios and media concentrations used and tested during optimisation. Number of cells is specified as a ratio, 50:50 for example, and media is specified as a percentage of total media volume.

| TT1 cells (number) | NCI-H441 cells (number) | DCCM-1 (%) | RPMI (%) |
| --- | --- | --- | --- |
| 50 | 50 | 100 | 0 |
| 50 | 50 | 50 | 50 |
| 50 | 50 | 0 | 100 |
| 11 | 1 | 100 | 0 |
| 11 | 1 | 50 | 50 |
| 11 | 1 | 0 | 100 |

Figure S3 - Concentrations of interleukin-8 (IL-8) (**A, B**) and interleukin-6 (IL-6) (**C, D**) in supernatants from co-cultures submerged in 100% TT1 growth media (DCCM-1) and in 50:50 ratio of TT1 media and NCI-H441 media (DCCM-1 + RPMI). TT1 and NCI-H441 monocultures in optimal conditions cell cultures in submerged conditions are also compared. Supernatants were taken every 24 hrs for a total of 144 hrs from both apical (**A, C**) and basal (**B, D**) regions of the cell culture and concentrations measured using an ELISA kit. Cells were seeded at 11:1 TT1: NCI-H441, respectively. Since media is replaced every 72 hrs, concentrations measured between 24 – 72 and 96 -144 hrs are cumulative. Data shown are n=3, ± SEM. Significance is noted using * when comparing TT1 monoculture with co-culture in 100% DCCM, **Ο** for TT1 monoculture vs co-culture in 50:50 media, □ when comparing TT1 monocultures with NCI-H441 monocultures, ●when comparing NCI-H441 monocultures and co-cultures in 50:50 media, ■ when comparing NCI-H441 monocultures and co-cultures in 100% DCCM-1 media. The number of significance symbols defines the level of significance; *=p<0.05, **=p<0.01, ***=p<0.001, ****=p<0.0001.

Figure S4 - Cell number (A) and viability (B) of TT1 and NCI-H441 co-cultures compared to TT1 and NCI-H441 monocultures, both in submerged conditions. Data calculated using trypan blue exclusion assay every 24 hrs after seeding. Co-culture was seeded at an 11:1 ratio, TT1: NCI-H441 respectively, and TT1 cells seeded at 5x10⁵ cells/ mL. Data shown are n=3, ±SEM. Statistical significance is noted using ***** when comparing TT1 monoculture with co-culture in 100% DCCM, **□** when comparing TT1 monocultures with NCI-H441 monocultures, **■** when comparing NCI-H441 monocultures and co-cultures. The number of significance symbols defines the level of significance; *=p<0.05, **=p<0.01.
